# Supplementary material for: Impaired in vitro Interferon-γ production in patients with visceral leishmaniasis is improved by inhibition of PD1/PDL-1 ligation
Source: PLoS Negl Trop Dis. 2022 Jun 24;16(6):e0010544. doi: 10.1371/journal.pntd.0010544 (PMC9262188; doi:10.1371/journal.pntd.0010544)
Supplement: S1 Table — PBMCs were isolated from whole blood as described in Material and Methods. The gating strategy is detailed in S2 Fig. Statistical differences were determined by a Mann-Whitney test. (DOCX) [file pntd.0010544.s001.docx]

Table S1: **PDL-1 expression on different monocyte subsets**

| **MFI PDL-1** | **VL patients** | **Control** | ***p* values** |
| --- | --- | --- | --- |
| **Classical** | 2951±1480 | 893±86 | <0.0001 |
| **Intermediate** | 5607±820 | 1269±218 | <0.0001 |
| **Non-classical** | 3451±425 | 1212±174 | 0.0061 |
