# Supplementary material for: Impaired in vitro Interferon-γ production in patients with visceral leishmaniasis is improved by inhibition of PD1/PDL-1 ligation
Source: PLoS Negl Trop Dis. 2022 Jun 24;16(6):e0010544. doi: 10.1371/journal.pntd.0010544 (PMC9262188; doi:10.1371/journal.pntd.0010544)
Supplement: S2 Table — PBMCs were isolated from whole blood as described in Material and Methods. The gating strategy is detailed in S2 Fig. Statistical differences between the 3 different subsets were determined by a Kruskal-Wallis test. (DOCX) [file pntd.0010544.s002.docx]

Table S2**: Comparison of** **PDL-1 expression between the different subsets**

|  | **Classical** | **Intermediate** | **Non-classical** | ***p* values** |
| --- | --- | --- | --- | --- |
| **MFI PDL1 VL patients** | 2951±1480 | 5607±820 | 3451±425 | 0.0082 |
| **MFI PDL1 controls** | 893±86 | 1269±218 | 1212±174 | 0.0572 |
