# Supplementary figures and images for: Impaired in vitro Interferon-γ production in patients with visceral leishmaniasis is improved by inhibition of PD1/PDL-1 ligation
Source: PLoS Negl Trop Dis. 2022 Jun 24;16(6):e0010544. doi: 10.1371/journal.pntd.0010544 (PMC9262188; doi:10.1371/journal.pntd.0010544)

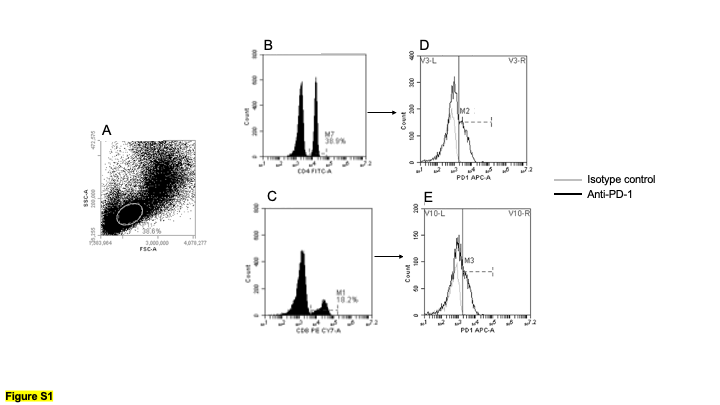

Supplement: S1 Fig — PBMCs were purified as described in Materials and Methods and the expression levels (Median Fluorescence Intensity [MFI]) of PD1 on CD4+ and CD8+ T cells were measured by flow cytometry. A. FSC and SSC of the lymphocyte gate (P1). B. CD4+ T cells in the lymphocyte gate (P1). C. CD8+ T cells in the lymphocyte gate (P1). D. PD1 (M2) on CD4+ T cells gate (M7). E. PD1 (M3) on CD8+ T cells gate (M1). (TIFF) [file pntd.0010544.s003.tiff]

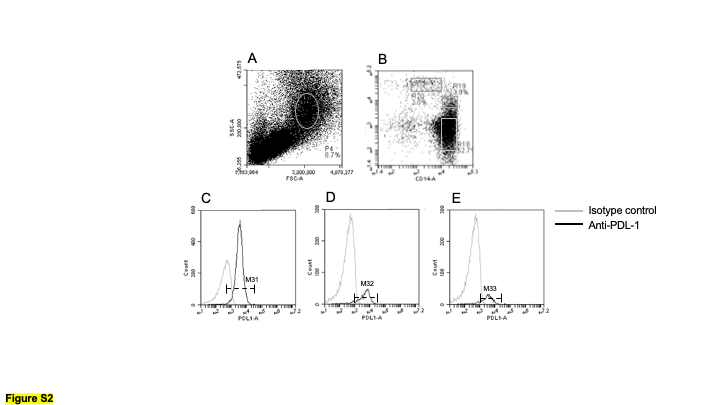

Supplement: S2 Fig — PBMCs were purified as described in Materials and Methods. PBMCs were stained with anti-human CD14APC, CD16FITC and PDL-1PE and the expression levels (Median Fluorescence Intensity [MFI]) of PDL-1 on the three subsets of monocytes were measured by flow cytometry. A. FSC and SSC of the monocyte gate (P4). B. Different monocyte subsets based on the expression levels of CD14 and CD16: classical (R18), Intermediate (R19) and Non-classical (R20) monocytes. C, D and E. PDL-1 MFI on the Classical (M31), Intermediate (M32) and Non-classical monocytes (M33). (TIFF) [file pntd.0010544.s004.tiff]

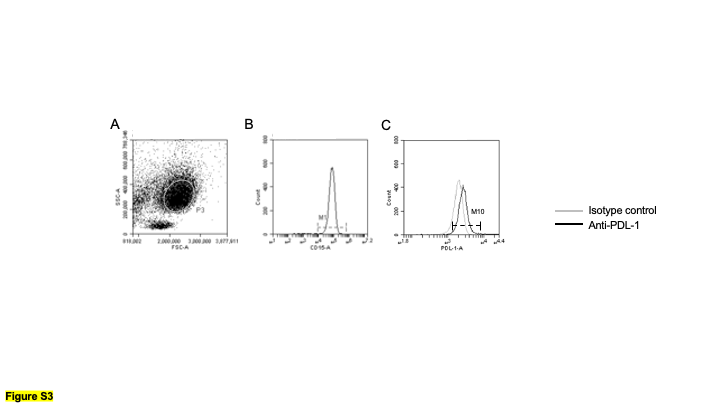

Supplement: S3 Fig — Neutrophils were purified as described in Materials and Methods and the expression of PDL-1 on neutrophils was measured by flow cytometry. A.FSC and SSC of the neutrophil gate (P3). B. CD15+ neutrophils in P3. C. PDL-1 MFI (M10) on neutrophils in M1. (TIFF) [file pntd.0010544.s005.tiff]
