# Supplementary material for: Impaired in vitro Interferon-γ production in patients with visceral leishmaniasis is improved by inhibition of PD1/PDL-1 ligation
Source: PLoS Negl Trop Dis. 2022 Jun 24;16(6):e0010544. doi: 10.1371/journal.pntd.0010544 (PMC9262188; doi:10.1371/journal.pntd.0010544)
Supplement: Attachment — Submitted filename: Response to reviewers Takeleetal210422.docx [file pntd.0010544.s006.docx]

We thank the reviewers for their positive and constructive comments. In agreement with their comments, we have now made substantial changes, highlighted in yellow, to our manuscript, as follows:

*Reviewer #3: About Figure 1: Are T cells stimulated? Is the difference of PD-1 expression in the presence or absence of SLA?*

No, the T cells were not stimulated; the MFI of PD1 on CD4^+^ and CD8^+^ T cells were measured directly *ex vivo*. The aim of this experiment was to assess the levels of PD1 on CD4^+^ and CD8^+^ T cells in the PBMCs isolated from the blood of VL patients at the time of diagnosis, directly *ex vivo*, without further stimulation; and compare it to those of healthy controls. This has been added in the text (page 11, line 185) and the legend of figure 1 (page 19, lines 345-348).

*About Figures 2 and 3: are they stimulated with SLA? Are there diferrences?*

No, the cells were not stimulated; the MFI of PDL-1 on the different monocyte subsets and the neutrophils cells were measured directly *ex vivo*. The aim of this experiment was to assess the levels of PDL-1 on monocytes and neutrophils from the blood of VL patients at the time of diagnosis, directly *ex vivo*, without further stimulation; and compare it to those of healthy controls. This has been added in the text (page 12, line 205 and page 19, lines 354-363).

*About figure 4: It is missing isotype control.*

This was an oversight. We have now added these important data as the new Figure 3B, and discussed them in the text (page 12, lines 220-222). Our results show no significant difference between the levels of IFNγ produced in response to SLA alone and those produced in response to SLA in the presence of the isotype control.

--------------------

<b>Results</b></br></br>
-Does the analysis presented match the analysis plan?</br>
-Are the results clearly and completely presented?</br>
-Are the figures (Tables, Images) of sufficient quality for clarity?</br></br>

Reviewer #1: The analysis presented by the author match with the analysis plan, and the results are clearly presented. The figures are of sufficient quality.

Reviewer #2: The experiments in this study are well chosen to provide information on the objectives.
Table 1 summarizes the clinical and haematological parameters of the VL patients and the non-infected control individuals.
*Could the authors distinguish between healthy individuals (not infected until then) and individuals that might have had an asymptomatic Leishmania infection before?* The healthy individuals who were recruited as control in our study were from a non-endemic area and had not travelled to an endemic area. Furthermore, they all tested negative by rk39. This information has now been added to the Material and Methods section, page 7, lines 112-113. We have also discussed this in the discussion section, page 14, lines 264-265.

*It might be beneficial to graphically depict the parasite grade of the different infected individuals for better insight into the variance in parasite load, which might correlate with the extent of the later analyzed PD-1 and PD-L1 expression as well as the IFN-y secretion.*

We tested for correlations between the parasite grades and the PD1 and PDL-1 MFI, but none of these correlations were significant. We have now added these results on page 12, line 213-214. We have previously shown that there is a clear negative correlation between parasite grade and IFN-γ was in our previous study (Takele, 2022).

*Fig. 1: Besides the already previously published PD-1 expression on CD4+ T cells (A), the authors now additionally determine PD-1 expression on CD8+ T cells (B), which is significantly increased in comparison to the healthy controls. 
Data in Panel 1a was already published previously, so why show it again, new donors? Please specify.*

These patients were specifically recruited for this study and the PD1 MFI has not been previously published.

*Please show the gating strategy that was applied to measure the PD-1 MFI of the T cell subpopulations. Did the authors set a gate on cell size (FSC/SSC), single cells, did they check for cell viability? Information should be provided in the material and methods section or graphically in the results section.*

The gating strategy is now shown as the new Figure S1. We did assess the viability of T cells, monocytes and neutrophils with healthy controls when we set up these experimental conditions, and the percentages of viable cells was >94%.

Fig. 2: As the binding of PD-L1 to PD-1 can elicit a T cell exhaustion phenotype which might explain a pronounced disease development. Thus, the authors measured the relative amount of PD-L1 that is expressed on blood monocytes.
For all three examined monocyte subtypes, there is a significant difference between PD-L1 expression in healthy controls and VL patients. *However, it’s not clearly stated why exactly those three monocyte subtypes were chosen for the analysis. What is their individual role in the recognition of Leishmania parasites or in activation of the adaptive immune system?*

We have now introduced more detail about the different monocyte subsets. The main aim of our study was to determine whether the impaired production of IFNγ can be improved by interfering with the PD1/PDL1 pathway. The characterisation of the different subsets of monocytes in VL patients is currently being addressed as part of another study in our laboratory. Importantly, the role of these different subsets in VL patients is poorly characterised. We have developed this on pages 11-12, lines 194-204.

*Can the authors confirm that these blood monocytes were (partially) infected with Leishmania?*

The monocytes were not infected with *Leishmania donovani* *ex vivo*. PDL-1 was measured on monocytes after gradient centrifugation, directly *ex vivo*, without further stimulation or infection with parasites (page 12, line 205).

*If not, what would be the trigger to make them express PD-L1.*

This is addressed in the discussion section, on page 13-14, lines 236-259.

*How would they get in contact with the parasites?*

We have not identified the mechanisms resulting in the upregulation of PDL-1 on monocytes, as we did not study the interactions of monocytes/parasites *in vitro*. According to the best of our knowledge, this has also not yet been defined on the different monocyte subsets during human visceral leishmaniasis. We do not know if it might be the direct interaction of host cells with parasites, or if it is mediated by the inflammatory environment as discussed on page 13-14, lines 236-259.

*Would it be an option to additionally investigate PD-L1 expression on macrophages from splenic aspirates, or even more specific from infected macrophages?*

No, it is not an option in our setting. Splenic aspirates are specifically performed for diagnosis purposes, a very small amount of tissue aspirate is collected that is smeared on a slide to be stained, the syringe is then rinsed in parasite medium for promastigotes growth. It is therefore not possible to obtain enough cells for research purposes. Furthermore, in this study we measured the production of IFNγ by whole blood cells and assess whereas interfering with the PD1/PDL-1 pathway improved the production of IFNγ by whole blood cells; therefore, it makes sense to identify cells in the blood that express PD1 and PDL-1. We recognise that in this specific study, we studied the role of PD1/PDL-1 blockade on IFNγ production in the blood and that we cannot exclude that this is different in other organs affected by VL. We have added a sentence to this effect, page 15, lines 297-300.

*Figure 2D and 2E are redundant to Fig 2A-C as this is just another combination of the graphs. This is not correct, please change accordingly and put all 6 datasets into one chart in order to show all relevant comparisons and significances.*

In agreement with this reviewer’s comment, we have now eliminated Figures 2D and E, we have also added new Tables S1 and S2 with all the relevant comparisons.

*Fig. 3: Here the authors show the PD-L1 expression by neutrophils. As in Fig 2, it is not clear if these neutrophils are (partially) infected with Leishmania in the blood, which would explain their activation.*

The neutrophils were not infected with *Leishmania donovani* *ex vivo*. PDL-1 was measured on neutrophils after dextran sedimentation, directly *ex vivo*, without any further stimulation or infection with parasites (page 12, line 211 and page 19, line 369).

*Once recruited to the affected organs (spleen, liver) neutrophils might fulfill their function as local drivers of an immune response but they might not get back into the blood stream.*

We recognise that in this specific study, we studied the role of PD1/PDL-1 blockade on IFNγ production in the blood and that we cannot exclude that this is different in other organs affected by VL. We have added a sentence to this effect, page 15, lines 297-300.

*The authors should also state in the figure legend or results text which surface markers were used for identifying the cells as neutrophils (CD15, CD16).* This information has been added in the new Figure S3.

Fig.4: Treatment of whole blood cells from VL patients with SLA in the presence of the anti-PD-1 mAB Nivolumab increases IFN-gamma secretion as shown by ELISA.
*How much SLA was used (concentration) and from which strain was it produced, please specify.*

This information has been added in the Materials and Methods section, page 7, lines 127 and page 8, lines 132-133.

*According to the methods section, cells were treated with 1µg Nivolumab. What was the total volume of the assay and/or the (approx.) number of cells treated?* This information has been added in the Materials and Methods section, pages 7-8, lines 125-131.

*The authors should also provide data on whole blood cells derived from healthy patients in this assay.*

We fail to understand the scientific rationale for this, as well as what the reviewer means by healthy patients. We have previously shown that whole blood cells from healed VL patients produce IFNγ in response to SLA (Takele, 2022), but that during active disease, this production of IFNγ was severely impaired. Our current aim was therefore to assess whether the impaired IFNγ response in response to SLA could be improved in VL patients during active disease by interfering with the PD1/PDL-1 interaction; but not to determine whether the restored IFNγ response in the whole blood assay can be further improved in healed patients.
*Reviewer #3: Figure 1: Please include the representative dot plot:*

The gating strategy and representative dot plots have been added as a new Figure S1.
*Figure 2: Please include the representative dot plot*

The gating strategy and representative dot plots have been added as a new Figure S2. *What is the difference in figure 2D? The median is very similar.*

In agreement with this reviewer’s comment, we have now eliminated Figures 2D and E, we have also added new Tables S1 and S2 with all the relevant comparisons.

*Please include the representative dot plot:*

The gating strategy and representative dot plots have been added as a new Figure S2.
*Figure 4: 
Is there a possibility to do a correlation of expression of PD1 versus IFNg production?*

We tested for correlations between the parasite grades and the PD1 and PDL-1 MFI, but none of these correlations were significant. We have now added these results on page 12, lines 213-214. We have previously shown that there is a clear negative correlation between parasite grade and IFN-γ was in our previous study (Takele, 2022).

*What is the level of PD-1 before and after the treatment with anti-PD1.*

We didn’t measure the levels of PD1 after adding anti-PD1 to the cultures, as the aim of our study was to assess whether interfering with the interaction of PD1 with PDL-1 can improve the production of IFNγ by whole blood cells. Our results clearly show that this interference results in increased IFNγ production.

--------------------

<b>Conclusions</b></br></br>
-Are the conclusions supported by the data presented?</br>
-Are the limitations of analysis clearly described?</br>
-Do the authors discuss how these data can be helpful to advance our understanding of the topic under study?</br>
-Is public health relevance addressed?</br></br>

Reviewer #1: The conclusion are supported by the data provided. Yet, study limitations are not so well described. The subject is of highly public health relevance and the authors have clearly emphasized the importance of the current study.

Reviewer #2: no
no
yes
yes

Reviewer #3: *It is missing intracellular citokines IFNg  on CD4 and CD8 T cells to see the impact of anti-PD1 in each population*. The main aim of our study was to assess whether interfering with the PD1/PDL-1 pathway would result in improved IFNγ production. However, we agree that identifying the cellular source of IFNγ would have been of interest. This has already been shown convincingly by Kumar *et al.* (Kumar, 2014). As mentioned previously, these patients are severely pancytopenic. We tried to do intracellular cytokine staining; however we could not accumulate enough events to do a meaningful analysis from 1 ml of blood. This particular experiment should be done with a significantly larger amount of blood, but we were not allowed to collect more blood from these severely ill patients.

--------------------

<b>Editorial and Data Presentation Modifications?</b></br><br/>
Use  this section for editorial suggestions as well as relatively minor modifications of existing data that would enhance clarity. If the only modifications needed are minor and/or editorial, you may wish to recommend “Minor Revision” or “Accept”. 

Reviewer #1: Minor revision.

Reviewer #2: -

*Reviewer #3: Figure 2 and 3 could be combined.*

In agreement with the reviewer’s comment, we have combined Figure 2 and 2 in a new Figure 2
--------------------

<b>Summary and General Comments</b></br></br>
Use  this section to provide overall comments, discuss strengths/weaknesses of the study, novelty, significance, general execution and scholarship. You may also include additional comments for the author, including concerns about dual publication, research ethics, or publication ethics. If requesting major revision, please articulate the new experiments that are needed.

Reviewer #1: The authors in this study investigated the expression levels of PD-1 on CD4 and CD8 T cells of visceral leishmaniasis patients, and of its ligand PDL-1 on monocytes and neutrophils. The authors have found that anti-PD-1 treatment of whole blood cells of VL patients increases the production of INFg.

General comments:

The article requires minor modification that can be addressed through revision. The study addressed the interruption of PD-1/PDL-1 pathway, but do not mention the other ligand PDL-2 which has been shown to contribute as well to the success of anti-PD-1 treatment. *It would be interesting to introduce PDL-2 as another ligand of PD-1 and discuss whether PDL-2 has been addressed in VL or not and the consequences.*

We have now addressed these points in the discussion, page 14, lines 266-273. To the best of our knowledge, no studies have addressed the role of PDL-2 in human visceral leishmaniasis. One study identified a role for PDL-2 in VL in the hamster model of VL, as they show that interfering with its pathway resulted in lower parasite load. We have now added these points in the discussion, page 14, lines 271-273.

Methods:
 *-       How SLA is produced? Could the authors describe on that as well?*

The detailed protocol that we used for the production of SLA has been added in the Materials and methods section, page 8, lines 132-145.

*-       What are the clones of the monoclonal antibodies used for FACS? This information should be in the paper.*

The different clones for the monoclonal antibodies have been in the Materials and methods section, page 8, lines 148-150.

*-       What is the clone of the commercial anti-PD-1 used for blocking assays? Is this information possible to add in the paper?*

This information has been added in the Materials and methods section, page 7-8, lines 128-129. *-       The authors inform that acquisition was performed on a BD Accuri B6, how many events were acquired per sample? How was the gating strategy used for the analyses?*

All the gating strategies have been added as supplementary Figures S1, S2 and S3.
*-       Why the authors did not use CD3 or CD45 in the panels to better define the cell types investigated?*

We used FSC/SSC and CD14 and CD16 for the monocytes in the PBMCs; CD4 and CD4 or CD8 for the T cells in the PBMCs; and CD15 for the purified neutrophils, all the gating strategies have now been added as supplementary Figures S1, S2 and S3.

*-       The authors should explain why they did not use the same antibody isotype of PD-1 as control for the antibody blocking assay. This is a critical part of the methods used in the paper.*

This was an oversight. We have now added these important data as Figure 3B, and discussed them in the text (page 12, lines 220-222). Our results show no significant difference between the levels of IFNγ produced in response to SLA alone and those produced in response to SLA in the presence of the isotype control
Figures:

*Table 1. should be reformatting.*
We have now reorganised and clarified Table 1
*Fig 1. It would be interesting to provide all the gating strategy used to define CD4 and CD8 T cells, and the other cell types. This information could be as supplementary material to the article. Did the authors observed a frequency difference as well?*

All the gating strategies have now been added as new Figures S1, S2 and S3. Differences in the frequencies of CD4 and CD8 were already published, not for this specific cohort of patients, but for a significantly larger cohort of VL patients in our previous publication (Takele, 2022), where we show that there are significantly less CD4 and CD8^+^ T cells at ToD.

*Do the authors imagine that these T cells are exhausted? It would be interesting to discuss this as well*

This point has been addressed in the introduction and the discussion, on page 6, lines 96-99 and pages 13-14, lines 247-255.
*The information on the markers to define each cell type evaluated should be in figure legends as well.*

This has been addressed in the legends of the new figures showing the gating strategies (Figures S1, S2 and S3).

*Fig 2. Do the authors have any data on the levels of PDL-1 of in situ macrophages from biopsies? It would be interesting for the discussion.*

We agree that it would be interesting to perform the same experiments with splenic aspirates. However, this is not an option in our setting: splenic aspirates are specifically performed for diagnosis purposes, a very small amount of tissue aspirate is collected that is smeared on a slide to be stained, the syringe is then rinsed in parasite medium for promastigotes growth. It is therefore not possible to obtain enough cells for research purposes. Furthermore, in this study we measured the production of IFNγ by whole blood cells and assess whereas interfering with the PD1/PDL-1 pathway improved the production of IFNγ by whole blood cells; therefore, it make sense to identify cells in the blood that express PD1 and PDL-1. We recognise that in this specific study, we studied the role of PD1/PDL-1 blockade on IFNγ production in the blood and that we cannot exclude that this is different in other organs affected by VL. We have added a sentence to this effect, page 15, lines 297-300.

*Fig 4. Do the authors have an idea if blocking affects the production of other cytokines that could be relevant for VL as well? I understand that the main hypothesis was drafted on the impairment of IFNg production ToD, but it would be scientifically relevant to show the data on other cytokines as well, such as IL-10, IL-4, IL-2 (bdl) and TNF.*

We agree with the reviewer that it would be interesting to assess other cytokines in the supernatants of the WBA. However, as stated by the reviewer, the scope of our study was a follow-up of the study we published previously (Takele, 2022) to assess whether interfering with PD1 might result in increased IFNγ production in the whole blood assay. Importantly, the ELISA were performed in Ethiopia, where we do not have access to a multiplex. We didn’t have enough supernatants to test for other cytokines.

*The authors could at least discuss how the interaction of PD-1/PDL-1 mechanistically contribute to IFNg impairment during VL*.

We have addressed this point in the discussion, pages 14-15, lines 274-282 , as follows: PD-L1/PD1 interaction is a powerful antagonist of TCR signal transduction, as well as CD28 and ICOS costimulatory signalling. This interaction results in impaired cytokine production and cell cycle arrest; as well as in the reduction of the transcription of Bcl-X_L_, a mitochondria transmembrane molecule with anti-apoptotic properties. The ligation of PDL-1 and PD1 abrogates the phosphorylation of phosphorylation of various signaling molecules, such as ERK, Vav, PLCγ, and PI3K (Arasanz, 2017 #3901)(Yokosuka, 2012 #3902). However, the mechanism of PD-1-mediated T cell inhibition are still poorly understood. A recent study showed that T cell suppression via PD-1 ligation is a result of the inactivation CD28 signaling by PD1 ligation (Hui, 2017 #3903).

Reviewer #2: -

Reviewer #3: It is missing important references
*- PD-1 Blockade Modulates Functional Activities of Exhausted-Like T Cell in Patients With Cutaneous Leishmaniasis*

This reference has been discussed on page 15, lines 283-284. *- Leishmania Parasites Drive PD-L1 Expression in Mice and Human Neutrophils With Suppressor Capacity
DOI: 10.3389/fimmu.2021.598943*

This reference has been discussed on page 14, lines 257-259. *- Immunotherapy using anti-PD-1 and anti-PD-L1 in Leishmania amazonensis-infected BALB/c mice reduce parasite load
DOI: 10.1038/s41598-019-56336-8*

This reference has been discussed on page 15, lines 286-288.

--------------------

PLOS authors have the option to publish the peer review history of their article (<a href="<https://journals.plos.org/plosntds/s/editorial-and-peer-review-process#loc-peer-review-history>" target="_blank">what does this mean?</a>). If published, this will include your full peer review and any attached files.<br><br>

If you choose “no”, your identity will remain anonymous but your review may still be made public.<br><br>

<b>Do you want your identity to be public for this peer review?</b> For information about this choice, including consent withdrawal, please see our <a href="<https://www.plos.org/privacy-policy>" target="_blank">Privacy Policy</a>.

Reviewer #1: Yes: Rafael de Freitas e Silva

Reviewer #2: No

Reviewer #3: No

References:

Takele Y, *et al*. Immunological factors, but not clinical features, predict visceral leishmaniasis relapse in patients co-infected with HIV. Cell Rep Med. 2022; 3:100487

Kumar, *et al.* *Leishmania* specific CD4^+^ T cells release IFNgamma that limits parasite replication in patients with visceral leishmaniasis, 214, PLoS NTD, 8:e3198
